# Optimizing targeted drug selection in combination therapy for patients with advanced or metastatic renal cell carcinoma: A systematic review and network meta‐analysis of safety

**DOI:** 10.1002/cam4.5504

**Published:** 2022-12-01

**Authors:** Ruiyang Xie, Jie Wu, Bingqing Shang, Xingang Bi, Weixing Jiang, Chuanzhen Cao, Aiping Zhou, Hongzhe Shi, Jianzhong Shou

**Affiliations:** ^1^ Department of Urology, National Cancer Center/National Clinical Research Center for Cancer/Cancer Hospital Chinese Academy of Medical Sciences and Peking Union Medical College Beijing China; ^2^ Department of Medical Oncology, National Cancer Center/National Clinical Research Center for Cancer/Cancer Hospital Chinese Academy of Medical Sciences and Peking Union Medical College Beijing China

**Keywords:** mTOR inhibitor, renal cell carcinoma, targeted agents, VEGF‐TKI

## Abstract

**Objective:**

For patients with advanced or metastatic renal cell carcinoma (RCC), the dose of targeted agents was recommended in combination with immune checkpoint inhibitors. We performed a network meta‐analysis to describe a categorized safety ranking profile and assess the adaptability of the combination options of targeted agents.

**Methods:**

The targeted agents refer to vascular endothelial growth factor tyrosine kinase inhibitors (VEGF‐TKIs) and mammalian target of rapamycin (mTOR) inhibitors. Randomized controlled trials comparing these drugs were enrolled in a Bayesian model network meta‐analysis.

**Results:**

Nineteen clinical trials with 11 treatments and 10,615 patients were included. For grade ≥ 3 adverse events (AEs), compared with placebo, lenvatinib plus everolimus showed worse safety than all other treatments except for lenvatinib (placebo vs. OR 0.23, 95% CI 0.07–0.78). Everolimus was generally the safest agent (OR 1.23, 95% CI 0.50–3.14). Sorafenib arose the least renal AEs (placebo vs. OR 0.85, 95% CI 0.06–11.64), whereas lenvatinib plus everolimus had the highest risk of renal toxicity (placebo vs. 0.17 95% CI 0.01–1.02). For gastrointestinal symptoms, everolimus was related to much lower toxicity than other agents. In the respiratory safety analysis, tivozanib (placebo vs. OR 0.15, 95% CI 0.07–0.31) and axitinib (OR 5.43, 95% CI 3.26–9.22) were the riskiest agents. In terms of hepatobiliary (placebo vs. OR 0.44, 95% CI 0.09–2.10) and hemotoxicity (placebo vs. OR 1.03, 95% CI 0.14–7.68) related AEs, lenvatinib was found to be the safest treatment compared to placebo.

**Conclusions:**

Everolimus, with the best safety of grade ≥ 3, gastrointestinal, and respiratory AEs, was more likely to be considered for combination therapies. Lenvatinib appears to be the safest for blood/lymphatic and hepatobiliary AEs. For patients with renal disorders, sorafenib arises the least renal toxicity AEs. This study will guide treatment options and optimize the trial design for advanced or metastatic RCC.

## Graphical abstract

We carried out a network meta‐analysis to provide a categorized safety ranking profile and assess the adaptability for combination choice of targeted therapies. Overall, everolimus with the best ranking of safety, was more likely to be selected for combination therapies. This study will guide treatment choice and optimize the future trial design for advanced or metastatic RCC.

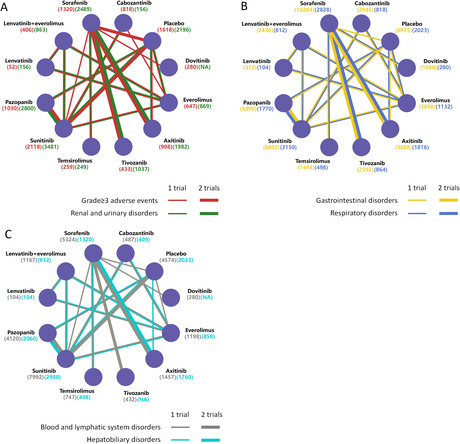

## 1 INTRODUCTION

The incidence rate of renal cell carcinoma (RCC) is increasing, and RCC accounts for approximately 3% of all cancers.^1^ Previous studies have revealed numerous mechanisms in the development and progression of RCC. Von Hippel–Lindau (VHL) gene inactivation can lead to the accumulation of hypoxia‐inducible factor, then cause overexpression of vascular endothelial growth factor (VEGF), which ultimately promotes neoangiogenesis and tumor growth.^2^ The VEGF tyrosine kinase inhibitors (VEGF‐TKIs) include sorafenib, sunitinib, pazopanib, axitinib, cabozantinib, tivozanib, and lenvatinib. These agents have become parts of the initial or alternative therapy for patients with advanced or metastatic RCC.^3^, ^4^ Furthermore, mTOR inhibitors, such as temsirolimus and everolimus, are therapeutic options other than VEGF‐TKIs therapies, but they have relatively lower efficacy and greater toxicity.^5^ Since the KEYNOTE‐426 study, immune checkpoint inhibitors (ICIs) combination has become by and large the standard of care for most patients with advanced RCC.^6^ By the European Association of Urology guidelines on RCC, monotherapies of targeted agents are only recommended to those patients who cannot receive or tolerate ICIs. The combination of ICI and targeted agent is preferred in the first‐line therapy of metastatic RCC, and the adverse events (AEs) of dual therapies are much more frequent than single agents. Several targeted agents including axitinib, cabozantinib, and lenvatinib have been considered candidates in combination therapy.^6^, ^7^, ^8^ Therefore, an evaluation of AEs of targeted therapies based on multiple organ systems is in need before the clinical trial is carried out. However, the safety of targeted agents has not been comprehensively analyzed to determine the potential of selecting them in combination with ICIs.

Advanced or metastatic RCC develops from localized RCC if early diagnosis is missed or treatment fails. Nephron‐sparing surgery and radical nephrectomy can prolong the survival of patients with localized or locally advanced RCC. However, the loss of nephrons may induce severe renal and urinary related AEs when systemic treatments are applied. A previous study showed that the downstream sequelae of chronic kidney disease might lead to excess mortality and poor survival outcomes among patients treated with radical nephrectomy.^9^ Previous network meta‐analyses partially compared targeted therapies for patients with advanced or metastatic RCC, but they did not include recent alternative treatments or describe toxicity‐related AEs (in accordance with renal toxicity, gastrointestinal toxicity, respiratory toxicity, hemotoxicity, and hepatic toxicity), which might be of particular concern to clinicians.^4^, ^10^ For now, the monotherapy of targeted agents is no longer recommended as the first‐line option. The efficacy of targeted agents needs to be reconsidered in the combination with immune checkpoint inhibitors, but the organ‐specific side effect profiles can help assess the adaptability of combination therapies. Thus, we conducted a network meta‐analysis of targeted agents for advanced or metastatic RCC to provide optimal options for clinicians.

## 2 METHODS

This network meta‐analysis was conducted based on the Preferred Reporting Items for Systematic Reviews and Meta‐Analyses (PRISMA) guidelines (Table S1).^11^ The Bayesian model was applied in this network meta‐analysis. The protocol was registered in the Prospective Register of Systematic Reviews (PROSPERO CRD42020212820). The local Institutional Review Boards of the Chinese Academy of Medical Science and Peking Union Medical College approved the meta‐analysis.

### 2.1 Database searching and study screening

The data extraction was performed by R. Xie and J. Wu, and any discrepancy was resolved by H. Shi and J. Shou. All the authors have reviewed the initial publication list. Articles in all languages published up to December 15, 2021, including those in PubMed, Embase, the CENTRAL registry of Cochrane Library, and ClinicalTrials.gov, were searched. The major search protocol consisted of the terms “renal cell carcinoma”, “VEGF‐TKIs”, and “mTOR inhibitors” (Table S2). Studies were included if outcomes grade ≥ 3 AEs, and toxicity‐related AEs of any grade (renal and urinary, hepatobiliary, gastrointestinal, blood and lymphatic system, and respiratory) were reported.

### 2.2 Criteria of study selection

The inclusion criteria of study selection were listed as follows:

1. Phase II/III randomized controlled trials with eligible published or unpublished results.
2. Trials with patients who were histologically diagnosed with advanced (stage III/IV/recurrent) or metastatic RCC.
3. Trials with an intervention arm including a VEGE‐TKI or an mTOR inhibitor.
4. Studies reporting at least one of the following clinical outcomes or AEs:
  (i) grade ≥3 AEs, defined as the sum of occurred AEs in grade 3–5.
  (ii) All AEs referred to in the Common Terminology Criteria for Adverse Events (CTCAE) version 5.0.^12^ AEs of any grade related to renal and urinary (proteinuria, creatinine increase, and renal failure), hepatobiliary (alanine aminotransferase increase and aspartate aminotransferase increase), gastrointestinal (abdominal pain, constipation, diarrhea, nausea, vomiting, and stomatitis), respiratory (dysphonia and dyspnoea), blood and lymphatic system (anemia, leukopenia, neutropenia, and thrombocytopenia), and other disorders (fatigue, weight decrease, rash, palmar‐plantar erythrodysesthesia, hypertension, and hypothyroidism).

Studies without the inclusion criteria were excluded. Other exclusion criteria were as follows:

1. Trials containing sequential or dose‐escalation treatment arms.
2. Trials in which treatments other than VEGF‐TKIs or mTOR inhibitors were involved.

### 2.3 Data extraction and assessment of bias risk

General characteristics including study ID, population sample size, age, sex, intervention arm, control arm, and reported AEs were extracted. Research data for outcomes were extracted from the intention‐to‐treat population. To avoid potential selective reporting bias, reported AEs of any grade were included, and those reported severe events only were excluded. The risk of bias in each study was assessed with the Cochrane risk of bias tool and shown in Figure S1.

### 2.4 Data synthesis and statistical analysis

We used the Bayesian random‐effects consistency model to conduct the network meta‐analyses of AEs. Odds ratios (ORs) with 95% confidence intervals (95% CIs) were used to describe rate outcomes for AE data. Second, node‐splitting analysis was conducted to test inconsistency in the network, in which *p* < 0.05 indicated significant inconsistency.^13^, ^14^ In addition, we assessed the ranking probability of the different agents for each AE by using the surface under the cumulative ranking curve (SUCRA) analysis.^15^ Finally, study heterogeneity was estimated by comparing the I^2^ values and mean difference if more than one trial existed.

To illustrate the sample size and number of trials, the network plots showing the toxicity‐related AEs were generated by the “GeMtc” and “rjags” packages in R 4.0.2 (https://www.r‐project.org/).^16^ The network meta‐analyses of AEs were completed in ADDIS software (version 1.16.6).^17^ To validate the reliability, network meta‐analyses of AEs were conducted in R as well. The number of chains was set to 3, whereas the thinning interval was 10, and the sample iterations parameter was adjusted to 100,000.

## 3 RESULTS

### 3.1 Characteristics of the enrolled trials

**FIGURE 1:**
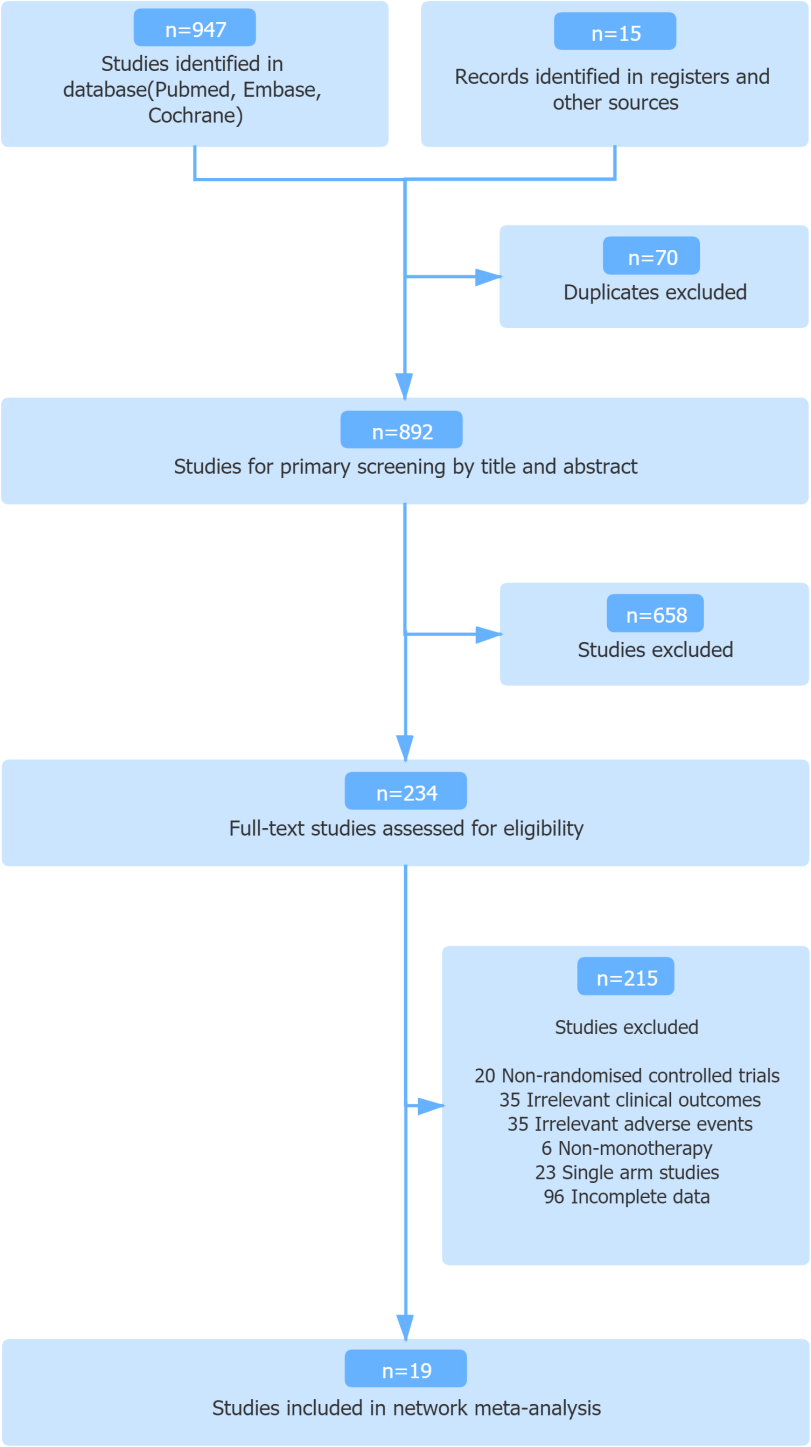
Study selection.

In total, 962 records from the database were identified and screened for title and abstract (Figure 1). Consequently, 19 randomized controlled trials and 11 treatments, including various VEGF‐TKIs and mTOR inhibitors (axitinib, cabozantinib, dovitinib, pazopanib, sorafenib, sunitinib, tivozanib, lenvatinib, lenvatinib+everolimus, temsirolimus, and everolimus), were enrolled in the study.^18^, ^19^, ^20^, ^21^, ^22^, ^23^, ^24^, ^25^, ^26^, ^27^, ^28^, ^29^, ^30^, ^31^, ^32^, ^33^, ^34^, ^35^, ^36^, ^37^, ^38^ The entire collection of studies is presented in Table 1, including the patient populations, group interventions, and reported AEs. Among the 19 studies with 10,615 patients in total, 19 reported grade ≥3 AEs and gastrointestinal disorders, 17 reported renal and urinary, respiratory, and blood and lymphatic system, and 13 reported hepatobiliary disorders.

**TABLE 1:** Baseline characteristics of studies included in the network meta‐analysis of patients with advanced or metastatic RCC

Study (Phase, ID)	Study centers (No)	Sample size (No); median age	Gender (male /female, %)	Group Intervention (Arm 1/arm 2/arm 3)	Reported adverse events
AXIS^19^ (III, NCT00678392)	294	361/362; NA	72.3/27.7	Axitinib 5 mg twice a day every 4 weeks/Sorafenib 400 mg twice a day every 4 weeks	Proteinuria, creatinine increased, alanine aminotransferase increased, aspartate aminotransferase increased, abdominal pain, constipation, diarrhea, nausea, vomit, stomatitis, dysphonia, dyspnea, anemia, leukopenia, thrombocytopenia, neutropenia, hypertension
TIVO‐1^22^ (III, NCT01030783)	86	260/257;59.0	72.3/27.7	Tivozanib 1.5 mg once a day every 4 weeks/Sorafenib 400 mg twice a day every 4 weeks	Proteinuria, renal failure, abdominal pain, constipation, diarrhea, nausea, vomit, stomatitis, dysphonia, dyspnea, anemia, hypertension
Sternberg et al^25^ (III, NCT00334282)	100	290/145;59.3	70.6/29.4	Pazopanib 800 mg once a day/Placebo	Proteinuria, alanine aminotransferase increased, renal failure, aspartate aminotransferase increased, abdominal pain, diarrhea, nausea, vomit, stomatitis, dyspnea, anemia, leukopenia, thrombocytopenia, neutropenia, hypertension
Hutson et al^29^ (III, NCT00474786)	128	259/253;59.85	75.2/24.8	Temsirolimus 25 mg once a week every 6 weeks/Sorafenib 400 mg twice a day every 6 weeks	Creatinine increased, renal failure, alanine aminotransferase increased, aspartate aminotransferase increased, abdominal pain, constipation, diarrhea, nausea, vomit, stomatitis, dysphonia, dyspnea, anemia, leukopenia, thrombocytopenia, hypertension
COMPARZ^31^ (III, NCT00720941)	227	557/553;61.1	73.2/26.8	Pazopanib 800 mg once a day/Sunitinib 50 mg once a day every 6 weeks	Proteinuria, creatinine increased, renal failure, alanine aminotransferase increased, aspartate aminotransferase increased, abdominal pain, constipation, diarrhea, nausea, vomit, stomatitis, dysphonia, dyspnea, anemia, leukopenia, thrombocytopenia, neutropenia, hypertension
Sub‐VEG108844^20^ (II, NCT01147822)	21	188/179;57.6	74.7/25.3	Pazopanib 800 mg once a day/Sunitinib 50 mg once a day every 6 weeks	Proteinuria, creatinine increased, renal failure, alanine aminotransferase increased, aspartate aminotransferase increased, abdominal pain, constipation, diarrhea, nausea, vomit, stomatitis, dysphonia, dyspnea, anemia, leukopenia, thrombocytopenia, neutropenia, hypertension
Hutson et al^30^ (III, NCT00920816)	126	192/96; NA	72.2/27.8	Axitinib 5 mg twice a day every 4 weeks/Sorafenib 400 mg twice a day every 4 weeks	Proteinuria, creatinine increased, renal failure, alanine aminotransferase increased, aspartate aminotransferase increased, abdominal pain, constipation, diarrhea, nausea, vomit, stomatitis, dysphonia, dyspnea, anemia, hypertension
S‐TRAC^26^ (III, NCT00375674)	115	309/306;57.9	73.3/26.7	Sunitinib 50 mg once a day every 6 weeks/Placebo	Proteinuria, creatinine increased, renal failure, alanine aminotransferase increased, aspartate aminotransferase increased, abdominal pain, constipation, diarrhea, nausea, vomit, stomatitis, dysphonia, dyspnea, anemia, leukopenia, thrombocytopenia, neutropenia, hypertension
ATLAS^32^ (III, NCT01599754)	106	363/361;58.0	73.2/26.8	Axitinib 5 mg twice a day every 4 weeks/Placebo	Creatinine increased, renal failure, alanine aminotransferase increased, aspartate aminotransferase increased, abdominal pain, diarrhea, nausea, vomit, stomatitis, dysphonia, dyspnea, hypertension
TIVO‐3^23^ (III, NCT02627963)	191	175/175;63.0	72.6/27.4	Tivozanib 1.5 mg once a day every 4 weeks/Sorafenib 400 mg twice a day every 4 weeks	Proteinuria, creatinine increased, renal failure, abdominal pain, constipation, diarrhea, nausea, vomit, stomatitis, dysphonia, dyspnea, anemia, hypertension
ASSURE^33^ (III, NCT00326898)	987	647/649/647; NA	67.4/32.6	Sunitinib 37.5 mg once a day every 4 weeks/Sorafenib 400 mg once or twice a day every 6 weeks/Placebo	Renal failure, diarrhea, nausea, anemia, leukopenia, thrombocytopenia, neutropenia, hypertension
TARGET^24^ (III, NCT00073307)	121	451/452;59.0	72.5/27.5	Sorafenib 400 mg once or twice a day/Placebo	Renal failure, constipation, diarrhea, nausea, vomit, hypertension
RECORD‐1^27^ (III, NCT00410124)	93	277/139; NA	77.4/22.6	Everolimus 10 mg once a day/Placebo	Creatinine increased, renal failure, abdominal pain, constipation, diarrhea, nausea, vomit, stomatitis, dyspnea, anemia, thrombocytopenia, hypertension
METEOR^28^ (III, NCT01865747)	205	330/328; NA	75.2/24.8	Cabozantinib 60 mg once a day/Everolimus 10 mg once a day	Alanine aminotransferase increased, aspartate aminotransferase increased, abdominal pain, constipation, diarrhea, nausea, vomit, dysphonia, dyspnea, anemia, hypertension
ASPEN^34^ (II, NCT01108445)	17	57/51;61.4	75.0/25.0	Everolimus 10 mg once a day on days 1 through 42 for each 42‐day cycle/Sunitinib 50 mg once a day on days 1 through 28 for each 42‐day cycle	Proteinuria, creatinine increased, renal failure, alanine aminotransferase increased, aspartate aminotransferase increased, abdominal pain, constipation, diarrhea, nausea, vomit, stomatitis, dysphonia, dyspnea, anemia, leukopenia, thrombocytopenia, neutropenia, hypertension
CABOSUN^35^ (II, NCT01835158)	488	78/79;63.0	78.3/21.7	Cabozantinib 60 mg once a day every 6 weeks/Sunitinib 50 mg once a day every 4 weeks	Proteinuria, creatinine increased, alanine aminotransferase increased, aspartate aminotransferase increased, abdominal pain, constipation, diarrhea, nausea, vomit, dyspnea, anemia, neutropenia, hypertension
Motzer et al^36^ (III, NCT01223027)	199	284/286; NA	75.8/24.2	Dovitinib 500 mg with 5 days on and 2 days off/Sorafenib 400 mg twice a day	Abdominal pain, constipation, diarrhea, nausea, vomit, stomatitis, dysphonia, dyspnea, anemia, hypertension
Motzer et al^37^ (II, NCT01136733)	37	51/52/50;61.3	72.8/27.2	lenvatinib 18 mg + everolimus 5 mg once a day/lenvatinib 24 mg once a day/everolimus 10 mg once a day	Proteinuria, creatinine increased, renal failure, alanine aminotransferase increased, aspartate aminotransferase increased, abdominal pain, constipation, diarrhea, nausea, vomit, stomatitis, dysphonia, dyspnea, anemia, thrombocytopenia, hypertension
CLEAR^38^ (III, NCT02811861)	200	357/357; NA	75.8/24.2	lenvatinib 18 mg + everolimus 5 mg once a day/Sunitinib 50 mg once a day every 4 weeks	Proteinuria, creatinine increased, alanine aminotransferase increased, aspartate aminotransferase increased, abdominal pain, constipation, diarrhea, nausea, vomit, stomatitis, dysphonia, dyspnea, anemia, thrombocytopenia, neutropenia, hypertension

Abbreviation: (NA = not applicable).

### 3.2 Network meta‐analysis in the consistency model

**FIGURE 2:**
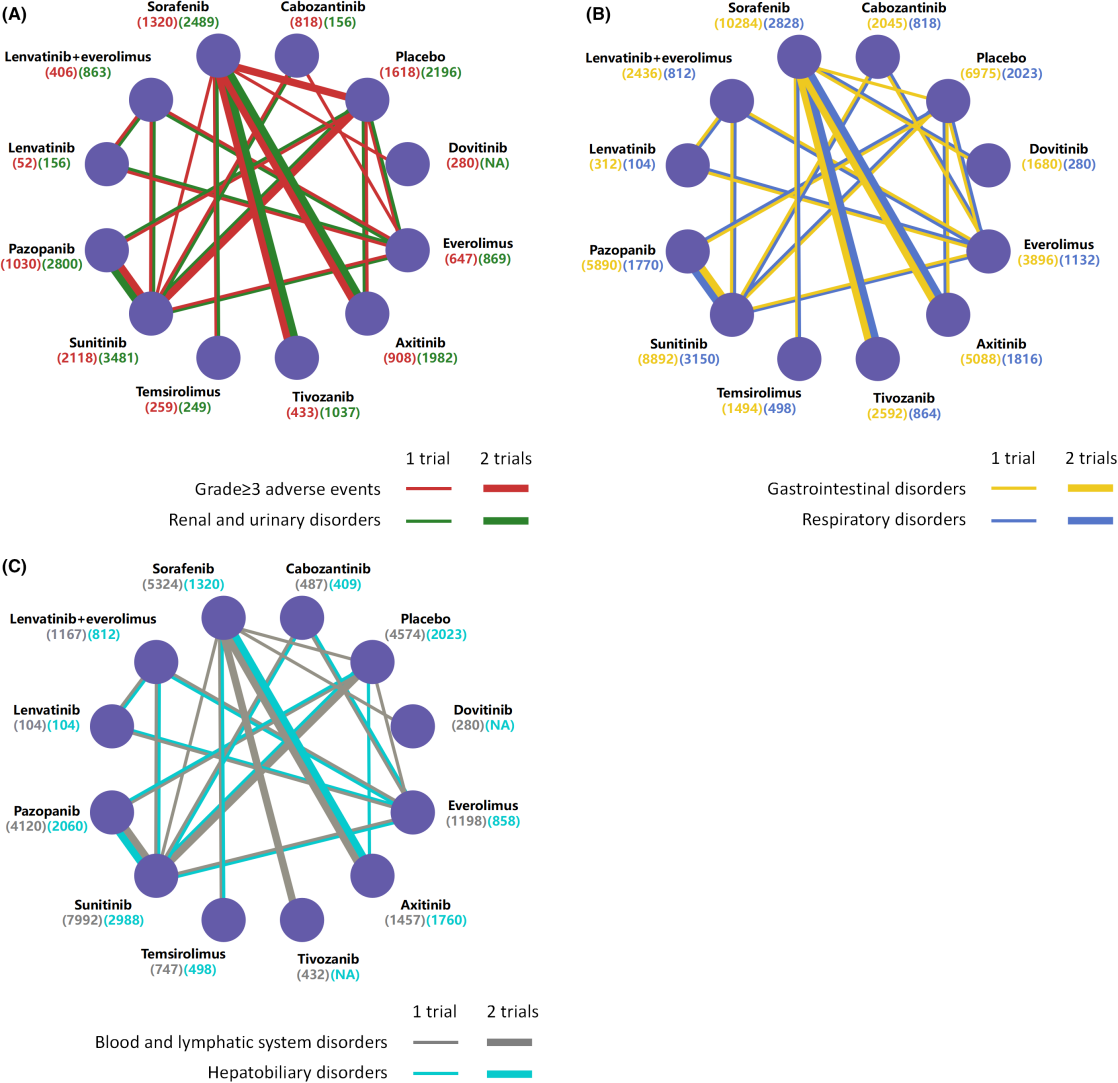
Network plots of comparisons on toxicity‐related adverse events (AEs) of treatments in patients with advanced or metastatic RCC. (A) Comparisons on grade ≥ 3 AEs and renal and urinary‐related AEs of any grade (B) Comparisons on gastrointestinal‐related adverse events and respiratory disorders‐related AEs of any grade (C) Comparisons on blood and lymphatic system‐related AEs and hepatobiliary of any grade. Each round node represents one single treatment. The total quantity of patients or AEs were shown in brackets. Each line represents a type of head‐to‐head comparison. The width of the lines is proportional to the number of trials comparing the connected treatments.

**FIGURE 3:**
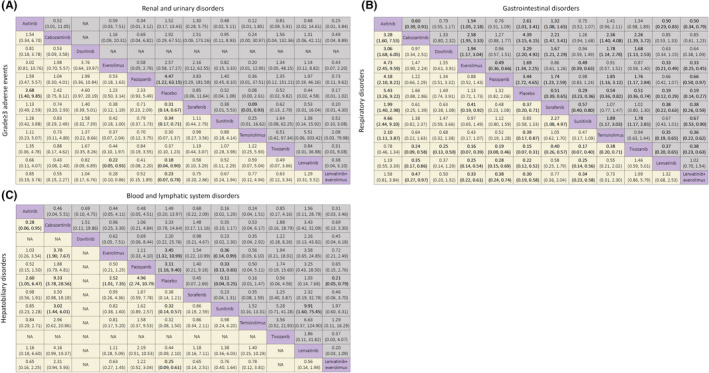
Pooled estimates of the network meta‐analysis. (A) Odds ratios (95% credible intervals) for grade ≥ 3 adverse events (AEs) (upper triangle) and renal and urinary‐related AEs (lower triangle). (B) Odds ratios (95% credible intervals) for gastrointestinal‐related AEs (upper triangle) and respiratory‐related AEs (lower triangle). (C) Odds ratios (95% credible intervals) for blood and lymphatic system‐related AEs (upper triangle) and hepatobiliary‐related AEs (lower triangle). Data in each cell are odds ratios (95% credible intervals) for the comparison of row‐defining treatment versus column‐defining treatment. Significant results are shown in bold.

Figure 2A–C show network plots for toxicity‐related AEs for 19 studies with 11 treatments. In terms of toxicity‐related AEs (Figure 3A–C), compared to placebo, lenvatinib plus everolimus (placebo vs. OR 0.23, 95% CI 0.07–0.78) showed more severe grade ≥3 AEs than other agents except for lenvatinib (placebo vs. OR 0.18, 95% CI 0.04–0.90). Overall, everolimus was proved to cause the least grade ≥3 AEs (vs. placebo OR 1.23, 95% CI 0.50–3.14). Among the targeted agents, temsirolimus caused the most severe renal AEs compared to placebo (placebo vs. OR 0.08, 95% CI 0.00–2.61), and lenvatinib plus everolimus was observed with a high risk as well (placebo vs. OR 0.17, 95% CI 0.01–1.02). In contrast to other VEGF‐TKIs, the OR of sorafenib was lower than 1 for renal and urinary AEs, suggesting that sorafenib might possess a lower risk of renal dysfunction. For gastrointestinal symptoms, lenvatinib was correlated with much higher toxicity than other agents (placebo vs. OR 0.19, 95% CI 0.12–0.29) but consistent with lenvatinib plus everolimus (OR 1.02, 95% CI 0.70–1.54). The safest agent for gastrointestinal symptoms appeared to be everolimus (vs. placebo OR 1.69, 95% CI 1.34–2.25). In terms of respiratory AEs, tivozanib (placebo vs. OR 0.15, 95% CI 0.07–0.31) and axitinib (vs. placebo OR 5.43, 95% CI 3.26–9.22) were the worst two agents. Notably, all the OR values of tivozanib and axitinib versus other treatments were statistically significant, whereas these two agents were consistent in the analysis. Again, everolimus was found to be a relatively safe agent in the analysis of respiratory symptoms (vs. placebo OR 1.13, 95% CI 0.72–1.73). As shown in Figure 3C, blood and lymphatic system AEs were also analyzed for each treatment. The AEs of sunitinib were the most severe (placebo vs. OR 0.11, 95% CI 0.04–0.25), while we found a similar risk in lenvatinib and placebo (placebo vs. OR 1.03, 95% CI 0.14–7.68). For hepatobiliary AEs, cabozantinib was found to be the treatment with the most hepatic toxicity (vs. placebo OR 9.33, 95% CI 3.78–28.56), whereas lenvatinib showed acceptable safety (placebo vs. OR 0.44, 95% CI 0.09–2.10). The profiles of each reported AE for pooled analysis are presented in Figure S2.

In addition, survival outcomes of progress‐free survival (PFS) and overall survival (OS) were also analyzed to describe the efficacy of targeted agents (Figure S4). For efficacy analysis, cabozantinib had the lowest statistically significant OR compared to placebo (vs. placebo OR 0.22, 95% CI 0.13–0.36) in PFS, as well as in OS (vs. placebo OR 0.69, 95% CI 0.30–1.40).

### 3.3 Rank probabilities

**FIGURE 4:**
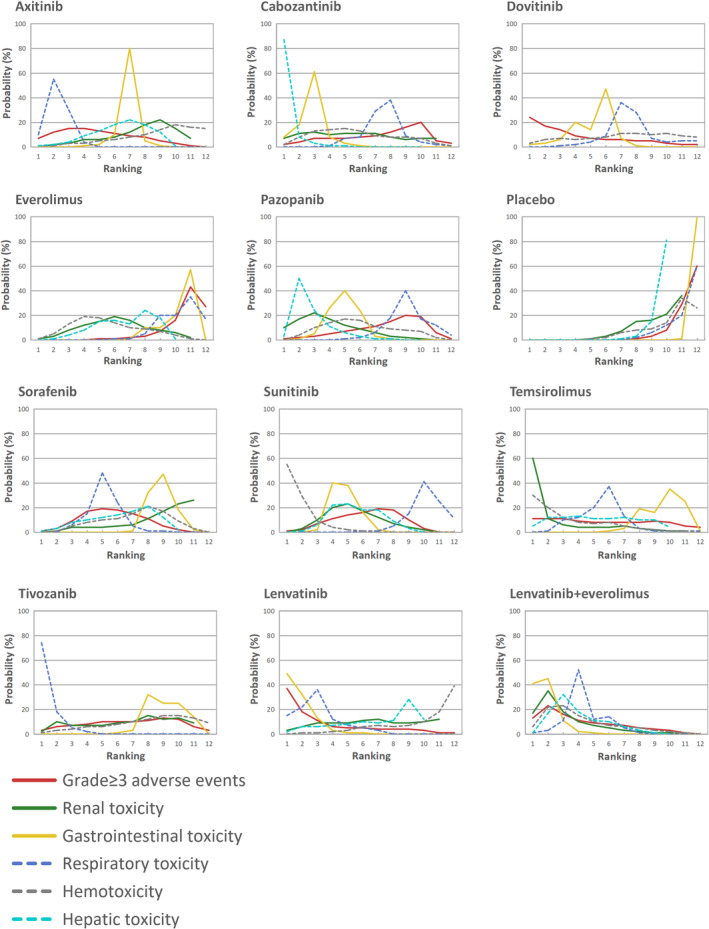
Bayesian ranking probabilities of comparable treatments on safety for patients with advanced or metastatic RCC. Profiles indicate the probability of each comparable treatment being ranked from worst to best on grade ≥ 3 AEs, renal and urinary, gastrointestinal, respiratory, blood and lymphatic system, and hepatobiliary in any grade. Ranking sources are described in Table S3.

Figure 4 shows the Bayesian ranking probabilities of toxicity‐related AEs among the 11 different treatments. The details of the ranking source are summarized in Table S3. All ranking probabilities were calculated based on the OR values mentioned previously. For grade ≥3 AEs, the ranking from the worst to the best was as follows: lenvatinib (ranking probability 37%), lenvatinib+everolimus (23%), dovitinib (14%), temsirolimus (9%), axitinib (13%), sorafenib (18%), sunitinib (19%), tivozanib (11%), pazopanib (20%), cabozantinib (20%), everolimus (43%), and placebo (60%). For toxicity‐related AEs, the treatments inducing the most severe toxicity were temsirolimus in renal toxicity (ranking probability 60%), lenvatinib in gastrointestinal symptoms (49%), tivozanib in respiratory dysfunction (74%), sunitinib in blood and lymphatic system (55%), and cabozantinib in hepatobiliary AEs (87%). The treatments that caused the least observed AEs were sorafenib in renal and urinary (23%), everolimus in gastrointestinal (57%), everolimus in respiratory (35%), lenvatinib in blood and lymphatic system (39%), and lenvatinib in hepatobiliary (28%). The ranking probabilities of efficacy in PFS and OS are shown in Figure S4C.

### 3.4 Sensitivity analysis

With a total of 18 trials, 8672 patients were included in the sensitivity analysis for grade ≥3 AEs (Figure S3). The ASSURE study^33^ was excluded due to the *p* value of 0.06 observed in the node‐split model analysis of sunitinib and placebo, suggesting possible inconsistency in direct and indirect comparisons. Consequently, no relevant deviations were observed compared to the original network meta‐analysis. However, sunitinib showed a higher probability of ranking in the fifth instead of ranking in the seventh.

### 3.5 Assessment of inconsistency

The inconsistency of AE data was estimated in a node‐splitting model and is presented in Table S4, where the *p* value <0.05 indicated a significant inconsistency between the direct effect and indirect effects. The table illustrated potential inconsistency in the renal toxicity network comparison of pazopanib versus placebo (*p* < 0.05), as well as in pazopanib versus sunitinib (*p* < 0.05). Furthermore, the hemotoxicity network comparison of everolimus versus placebo (*p* < 0.05). The remaining available comparisons showed minimal inconsistency with *p* > 0.05.

## 4 DISCUSSION

In the network meta‐analysis of VEGF‐TKIs and mTOR inhibitors, the overall results suggest the following:

1. Compared to other targeted drugs, lenvatinib plus everolimus and lenvatinib were correlated with more general severe AEs.
2. Considering all categories of AEs, everolimus appeared to be the safest treatment for combination therapy.
3. Temsirolimus and lenvatinib plus everolimus caused the most severe renal and urinary AEs among mTOR inhibitors and VEGF‐TKIs.

Before the great efficacy of ICIs for advanced and metastatic RCC was proved by scholars, targeted agents such as VEGF‐TKIs and mTOR inhibitors had been regarded as the standard of care.^39^ However, combination treatments of ICI and kinase inhibitor (e.g., pembrolizumab plus axitinib) were found to provide better survival outcomes than monotherapy for patients with metastatic RCC.^6^, ^40^ Theoretically, the combination of ICIs and targeted agents might cause more severe AEs than monotherapy, which should be fully estimated before the systemic treatment. In addition to general AEs, particular organ dysfunction might be frequent in patients with metastatic RCC. Surgical approaches, including nephron‐sparing surgery and radical nephrectomy, are important treatments in RCC, but the loss of nephron can lead to a greater burden in the form of systemic therapy for kidney issues. To individually optimize the appropriate combination therapies, the selection of targeted agent candidates in clinical trials needs safety assessment in particular systems.

Various targeted agents have been approved by the FDA and applied in patients with metastatic RCC. Because the efficacy of targeted agent monotherapies was interfered with by multiple mechanisms, recently targeted agents were designed to overcome these limitations. For example, the mesenchymal to epithelial transition factor (MET) and hepatocyte growth factor pathways have been demonstrated to play a role in resistance to VEGFR inhibitors such as sunitinib. Simultaneous inhibition of VEGFR and MET might overcome VEGFR inhibitor resistance and reinduce the antitumor effect,^41^ that is, one of the fundamental mechanisms of cabozantinib. Cabozantinib was combined with nivolumab in the CheckMate 9ER study, and higher rates of treatment‐related AEs were observed in the combination therapy arm than sunitinib alone for any grade and grade ≥3 (96.6% vs. 93.1%, and 60.6% vs. 50.9%, respectively).^8^ Furthermore, as reported in CLEAR study,^38^ lenvatinib plus pembrolizumab and lenvatinib plus everolimus showed worse safety than sunitinib. In our study, lenvatinib plus everolimus is associated with more severe toxicity‐related AEs than other VEGF‐TKIs, and kidney injuries occur more frequently in patients who underwent radical nephrectomy. Thus, a complete assessment is necessary for patients with renal dysfunction who plan to receive lenvatinib plus everolimus. In the safety analysis, everolimus was ranked in the eleventh for grade ≥3 AEs, the sixth for renal and urinary, the eleventh for gastrointestinal and respiratory, the fourth for blood and lymphatic system, and the eighth for hepatobiliary among the worst to the best. In general, everolimus seemed to be an ideal drug for combination therapy with acceptable safety. In the recently updated results of the CheckMate 025 study, treatment‐related AEs of any grade that occurred among patients in the everolimus arm were as follows: skin, 38.3%; gastrointestinal, 21.7%; hepatic, 7.8%; endocrine, 2.8%; renal, 9.1%; and pulmonary, 17.6%.^42^

### 4.1 Strengths and implications

Compared with the reported network meta‐analyses of treatments for patients with advanced or metastatic RCC,^4^, ^5^ this network meta‐analysis has several strengths. Combining mixed types of drugs in a single meta‐analysis without knowing the individual safety is not recommended because this might damage the consistency of the entire analysis. To better describe the safety of each treatment, our study established comparisons among all common VEGF‐TKI and mTOR inhibitor monotherapies and combination therapies, excluding sequential therapies and mixed therapies. As this study comprehensively analyzed the most recent versions of results and previously unpublished data, potential mistakes caused by various combinations of treatments were avoided. Furthermore, previous network meta‐analyses have tended to report AEs in terms of all grade 3/4 events or individual events, so these studies lacked categorical comparisons. Knowledge of common AEs can lead to early investigations and recognition of symptoms, which is highly significant to clinicians. This can help with the necessary drug dose reduction. In the immuno‐oncology era of RCC, combining VEGF‐TKIs with anti‐programmed death 1 (PD‐1) monoclonal antibodies or anti‐programmed death‐ligand 1 (PD‐L1) antibodies has been found to provide better efficacy than VEGF‐TKI monotherapy. However, a higher incidence of immune‐related AEs was also noted, indicating the combination of VEGF‐TKIs and ICIs might lead to more severe organ‐level effects. Therefore, our study identified AEs by classifying different AEs according to the systems they affect, providing categorical options and guidance to minimize drug‐related toxicity.

### 4.2 Limitations

The present study had several limitations. First, those trials that reported only severe AEs were excluded to minimize heterogeneity and reporting bias. However, this potentially decreased the total sample size and limited a complete analysis of AEs. Second, first‐line and second‐line treatments were analyzed comprehensively in this study to achieve the maximal network and sample size; thus, inevitable heterogeneity might exist among the trials. Third, inconsistency of analysis was observed in some comparisons (renal toxicity and hemotoxicity), which might be related to the difference in the number of patients enrolled in the studies. Finally, most trials failed to identify standardized methods for estimating treatment‐related AEs. The conclusions of this study may change if these missing data are updated in the future.

## 5 CONCLUSIONS

Based on this network meta‐analysis, everolimus, the mTOR inhibitor, could be the generally safest drug for combination therapies in grade ≥ 3, gastrointestinal, and respiratory AEs, while the VEGF‐TKI as lenvatinib appears to be the safest in terms of hepatobiliary and blood/lymphatic AEs. For patients with renal disorders, sorafenib causes the least renal toxicity AEs. Lenvatinib appears to be the worst in terms of grade ≥ 3 AEs. We also found that the safety of each treatment differed according to the category of toxicity‐related AEs, and patients should be assessed for specific underlying diseases before the doses of VEGF‐TKIs and mTOR therapies are chosen. These findings could guide treatment options and optimize the trial design for advanced or metastatic RCC.

## AUTHOR CONTRIBUTIONS

**Ruiyang Xie:** Conceptualization (supporting); data curation (lead); formal analysis (lead); methodology (lead); software (lead); writing – original draft (lead); writing – review and editing (lead). **Jie Wu:** Data curation (equal); formal analysis (equal); investigation (equal); methodology (lead); software (equal); writing – original draft (equal); writing – review and editing (equal). **Bingqing Shang:** Formal analysis (supporting); software (supporting); writing – original draft (supporting); writing – review and editing (supporting). **Xingang Bi:** Conceptualization (equal); supervision (equal); writing – original draft (supporting); writing – review and editing (supporting). **Weixing Jiang:** Methodology (supporting); software (supporting); writing – original draft (supporting); writing – review and editing (supporting). **Chuanzhen Cao:** Formal analysis (supporting); methodology (supporting); writing – original draft (supporting); writing – review and editing (supporting). **Aiping Zhou:** Conceptualization (equal); supervision (equal); writing – original draft (supporting); writing – review and editing (supporting). **Hongzhe Shi:** Conceptualization (equal); methodology (equal); project administration (equal); supervision (equal); writing – original draft (equal); writing – review and editing (equal). **Jianzhong Shou:** Conceptualization (lead); project administration (lead); supervision (lead); writing – original draft (equal); writing – review and editing (equal).

## FUNDING INFORMATION

None.

## CONFLICT OF INTEREST

None declared.

## PATIENT CONSENT STATEMENT

Not required.

## ETHICS APPROVAL STATEMENT

The protocol was registered in the Prospective Register of Systematic Reviews (PROSPERO CRD42020212820). The Institutional Review Boards of the Chinese Academy of Medical Science and Peking Union Medical College approved the study.

## Supporting information


Appendix S1

## Data Availability

The datasets supporting the conclusions of this article are included within the article and its additional file.
